# Discontinuity Detection in the Shield Metal Arc Welding Process

**DOI:** 10.3390/s17051082

**Published:** 2017-05-10

**Authors:** José Alberto Naves Cocota, Gabriel Carvalho Garcia, Adilson Rodrigues da Costa, Milton Sérgio Fernandes de Lima, Filipe Augusto Santos Rocha, Gustavo Medeiros Freitas

**Affiliations:** 1 School of Mines, Federal University of Ouro Preto (UFOP), Morro do Cruzeiro, 35400-000 Ouro Preto, Brazil; adilson@em.ufop.br; 2 Instituto Tecnológico Vale (ITV)—Avenida Juscelino Kubitschek, 31, Bauxita, 35400-000 Ouro Preto, Brazil; gabrcg@gmail.com (G.C.G.); f.rocha41@gmail.com (F.A.S.R.); gustavo.medeiros.freitas@itv.org (G.M.F.); 3 Institute for Advanced Studies (IEAv-CTA), 12228-970 São José dos Campos, SP, Brazil; miltonsflima@gmail.com

**Keywords:** support vector machine, artificial neural network, shielded metal arc welding, sensory fusion

## Abstract

This work proposes a new methodology for the detection of discontinuities in the weld bead applied in Shielded Metal Arc Welding (SMAW) processes. The detection system is based on two sensors—a microphone and piezoelectric—that acquire acoustic emissions generated during the welding. The feature vectors extracted from the sensor dataset are used to construct classifier models. The approaches based on Artificial Neural Network (ANN) and Support Vector Machine (SVM) classifiers are able to identify with a high accuracy the three proposed weld bead classes: desirable weld bead, shrinkage cavity and burn through discontinuities. Experimental results illustrate the system’s high accuracy, greater than 90% for each class. A novel Hierarchical Support Vector Machine (HSVM) structure is proposed to make feasible the use of this system in industrial environments. This approach presented 96.6% overall accuracy. Given the simplicity of the equipment involved, this system can be applied in the metal transformation industries.

## 1. Introduction

The Shielded Metal Arc Welding (SMAW) process is a simple, low cost and suitable way of joining most metals and alloys commonly used in industry [1]. Due to these characteristics, SMAW is the chief joining process used in the developing countries, such as India, China and throughout Latin American [2]. As a shortcoming, however, it is a predominantly manual process with low duty cycle.

In order to increase repeatability and improve the weld bead quality, Lima II and Bracarense [3] proposed robotic automation of the process, considering the Toll Center Point (TCP) variation during trajectory generation. When replacing manual devices with automatic ones, it is necessary to implement a controller based on the process knowledge. For SMAW this involves a multidisciplinary approach with complex pairing of input-output variables [4]. Therefore, instrumentation and modeling are key issues for the successful implementation of such controllers [5].

In industry, non-destructive techniques (NDT) predominate in the detection of discontinuities for quality control. Krolczyk et al. [6] discussed some recent trends in NDT for weld diagnosis, and assessed the use of Infinite Focus Measurement Machine, X-ray and Computed Tomography for topographic inspections. Welding process monitoring is nowadays one of the most important industrial research topics. Currently, most of the industrial systems used for monitoring welding processes adopt sensors with fast dynamics and relatively low cost, such as photodiodes, pyrometers and microphones [7]. Photodiodes are associated with filters to limit the signals to the light wavelengths of interest. Their main disadvantage is the low efficiency in identifying small defects. Pyrometers sample the temperature of the weld pool. However, they have limited capacity for discontinuity detection. The microphone is very sensitive to the ambient noise in an industrial environment. In contrast, the sound emitted through the interaction of the arc with the workpiece is a promising signal for the qualification of the weld bead. This signal can be filtered using the Blind Source Separation (BSS) technique [8], or a sensory fusion can be performed by associating it with another process signal [9]. A recent research proposes the use of ultrasonic transducer to diagnose incomplete fusion and crack formation faults in TIG processes [10]. Since this sensor operates at high frequencies, it is less sensitive to disturbances. However, its application in conventional NDT has some operational restrictions [11]. It is worth mentioning that visual sensors, due to cost reduction, are becoming more common for monitoring welding processes. The visual detection accuracy strongly depends on the pattern recognition algorithm [7].

Different Artificial Neural Network (ANN) topologies have been extensively explored to predict the weld bead geometry (width, height and penetration). Andersen et al. [12] conducted one of the pioneering studies with ANN in arc welding processes. Their work was accomplished using a Back Propagation Artificial Neural Network (BPANN) to predict the weld bead geometries from the Tungsten Inert Gas (TIG) process parameters. Later, those researchers explored the application of ANN in Variable Polarity Plasma Arc Welding [13]. They proposed the use of some ANN structures to locate undercut discontinuities based on scanned weld profiles and to relate the welding parameters with weld bead geometry. Nagesh and Datta [14] employ BPANN to predict the bead geometry from SMAW parameters. Further investigation on arc weld bead imperfection prediction with ANN has been done. Sterjovski et al. [15] described results obtained with two ANN models to assist the Flux-Cored Arc Welding process. First, they implemented a BPANN structure for predicting diffusible hydrogen based on process parameters. Then, they proposed a Probabilistic ANN to predict the probability of Hydrogen Assisted Cold Cracking induced by a gap on a steel sheet. Mirapeix et al. [16] investigated a classification of incomplete fusion cases in TIG welding processes by the spectroscopic analysis of the plasma spectra, using feature extraction from spectrometer measurements by Principal Component Analysis and the classification of the welds with BPANN. Afterward, the authors reported some advantages of using Sequential Floating Forward Selection for feature extraction [17]. Kumar et al. [18] presented the use of a Digital Storage Oscilloscope (DSO) to evaluate the performance of SMAW power sources and to rank the skill of welders using Self-Organizing Map neural network models. The main challenge for the use of DSO is its limited sampling rate, which is inadequate for variables that require high sampling rates.

Ancona et al. [19] presented an algorithm that compares the plasma temperature of the laser welding process with bounded lines constructed during the self-learning mode, which was later implemented for on-line TIG quality monitoring [20]. Another computational intelligence technique highlighted in the last decade is the Support Vector Machine (SVM). Among the advantages of this classifier with respect to ANN, one may mention the shorter classifier training time and higher generalization capacity. Huang et al. [21] investigated the SVM classification model for porosity defect detection in TIG processes. They reported the use of empirical mode decomposition for feature extraction of the signal from a spectrometer, and the use of a genetic algorithm to select the classifier parameters. However, there is no record in the literature of the use of any pattern recognition techniques, including SVM, for discontinuity detection in the SMAW process.

This work proposes a new methodology for discontinuity detection in weld beads during SMAW. The experimental configuration consists of sampling the air-borne and solid-borne acoustic emissions of the arc interaction with the workpiece. The signals obtained experimentally are filtered through signal spectrum Average Downsampling and Wavelets for features extraction. The spectrum of the audible signal from the desirable weld bead, shrinkage cavity and burn through discontinuities are discussed. The classifiers adopted for discontinuity detection are the SVM and ANN, and the obtained results are presented. A novel HSVM structure with sensorial fusion is presented, allowing the application of the proposed technique in industrial environments. Due to the equipment simplicity, as well as its low cost, the results of this work can be reproduced in other laboratories and applied to the industry.

## 2. Materials and Methods

### 2.1. Experimental Setup

**Figure 1:**
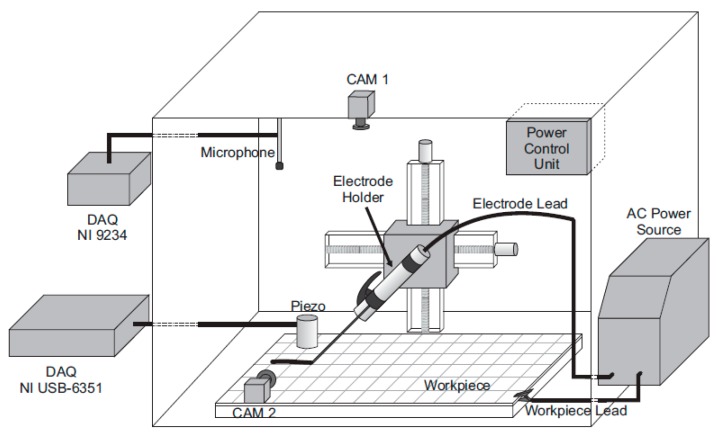
Experimental setup. The workstation is composed of a measuring system, constant current AC power source and electrode holder attached to a planar robot.

The workstation is composed of a measuring system, a constant current AC power source of up to 140 A and an electrode holder attached to a planar robot. A diagram of the workstation is shown in Figure 1. The measurement system consisted of a microphone (40PH, G.R.A.S., Holte, Denmark) and an ultrasonic transducer (H2-SE-20/0° 2 MHz piezoelectric, Eddytronic, Mairiporã, Brazil). The audible (air-borne) signal is sampled by the microphone via the NI 9234 data acquisition device (DAQ, National Instruments, Austin, TX, USA) at 50 kHz. The omni-directional microphone is positioned within the workstation at a distance of 550 mm from the workpiece surface. Acoustic emission monitoring (solid-borne) is performed according to the ASTM E749 standard, employing the piezoelectric sensor, which is rigidly attached to the steel plate, using the NI USB-6351 DAQ at 1.2 MHz. Due to the high sampling volume, the signal is only acquired during the discontinuity period. Two cameras recorded images of the arc and the melting pool.

**Figure 2:**
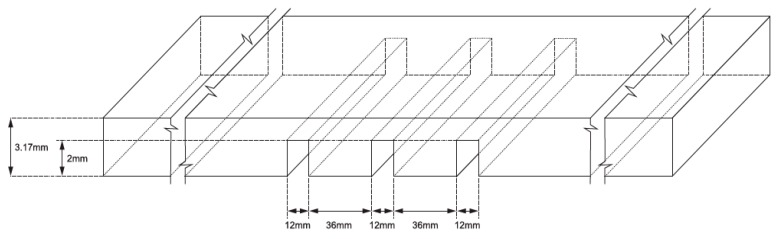
Base metal dimensions. Transversal slots with 12 × 2 mm on the lower surface of the steel sheet induce bead discontinuities.

Rutile electrodes (E6013, ESAB, Contagem, Brazil with 2.5 mm diameter were used to deposit the weld beads on a low carbon steel sheet of 1/8 inch (3.17 mm) thickness. Transversal slots with 12 × 2 mm on the lower surface of the steel sheet, as shown in Figure 2, induce bead discontinuities.

To look for discontinuity repeatability during the experiments, the slots have equal dimensions. Similar to the methodology proposed in [8], the slots were implemented in such a way that the base metal suffers burn through discontinuities when the arc covers that area. The slots also introduce shrinkage cavity discontinuities during the welding experiments.

The welding was performed in the flat position in pull mode with currents of 70 and 75 A, welding speed of 4 mm/s, feed speed of 4.2 mm/s and an electrode angle of −25°. A compound helped to establish the arc at the beginning of the process [3]. In order to prevent contamination, the base metal surface was cleaned. A planar robot with prismatic joints kept the welding and feed rates constant during the movement of a stringer bead pass [22].

### 2.2. Feature Extraction

A feature can be defined as a quantitative representation of a phenomenon observed in the sampled data [23]. The process of searching for features in the dataset is a fundamental step in the recognition of patterns and machine learning [24]. The first step for features extraction consists on data segmentation, e.g., dividing the signal into short-term frames, according to the selection of desired features. The signal sampled by the microphone was segmented into 196 frames, and the signal sampled by the piezoelectric sensor was segmented into 86 frames.

**Figure 3:**
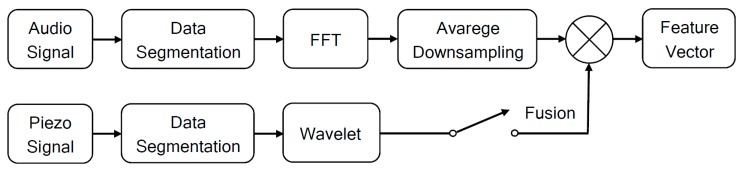
Block diagram of proposed method for feature extraction.

The Fast Fourier Transform (FFT) with Average Downsampling in sequence is applied to the segmented air-borne signals database for feature extraction. The Wavelet transform extracts feature from solid-borne signals. Considering other papers from literature, signal processing techniques such as FFT and Wavelet have been applied to monitor welding processes [25,26,27]. However, there are no reports of the use of Average Downsampling for signal processing during welding. The methodology used in the paper is shown in Figure 3.

**Figure 4:**
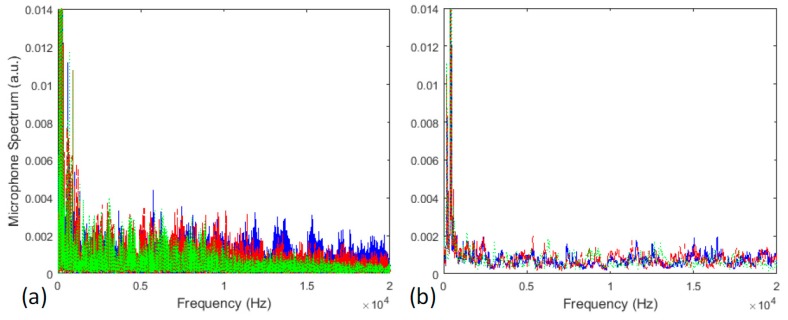
Preprocessing audible sound of a weld bead, (**a**) spectral signal derived from FFT; (**b**) spectral signal after average downsampling. Burn through audio signal (in blue), shrinkage cavity audio signal (in green) and desirable weld audio signal (in red).

Each short-term frame of the audible signal contains 80,000 samples, which were processed by the signal spectrum average value obtained by FFT, according to Equation (1):

(1) $$\mu \left(i\right)=\frac{W}{F}{\left[\sum_{\left(i-1\right)\left(\frac{F}{W}\right)+1}^{i\left(\frac{F}{W}\right)}x\left(i\right)\right]}_{i=1}^{W},$$

where μ(i) is the vector with the 5000 sliding windows averages, x(i) is the signal FFT, F is the number of the short-term frame samples and W is the number of sliding windows. The FFT algorithm computes the Discrete Fourier Transform (DFT) of data sequence and converts the time domain signal into a frequency domain representation. The main advantage of the FFT transform is the processing speed. The Average Downsampling smooths the spectrum of audible signal and reduces its dimensionality (Figure 4). Considering the frequency distribution, the burn through discontinuity signal presents high-order harmonics. This result is opposite to the ones observed in laser welding [8]. The difference in spectrum distributions should be related to the arc that opens and transfers the material to the contours of the burn through discontinuity, resulting in sound pressure fluctuations in SMAW.

**Figure 5:**
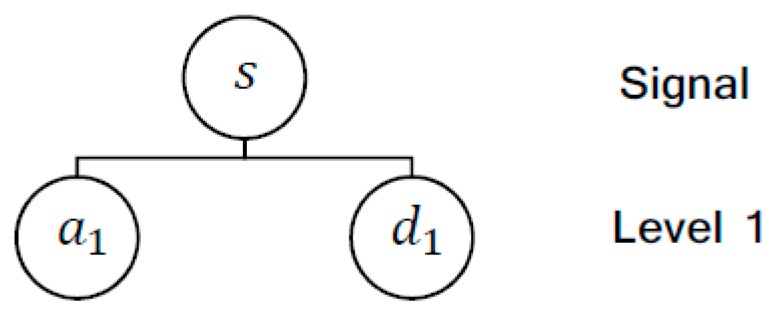
Wavelet decomposition tree for one dimension, divide the signal (s) into approximation ($a_{i}$) and detail ($d_{i}$) coefficients at each level.

In contrast, the acoustic emission signal contains 2 M samples for the same time interval. This signal was decomposed by Wavelet in the first level by the Daubechies 4 (db4) mother-wavelet. The Wavelet transform divides the signal into approximation and detail coefficients at each level of decomposition, as shown in Figure 5. This decomposition consists of the application of low-pass and high-pass filters, which result respectively in approximations and details.

### 2.3. Classifiers

**Figure 6:**
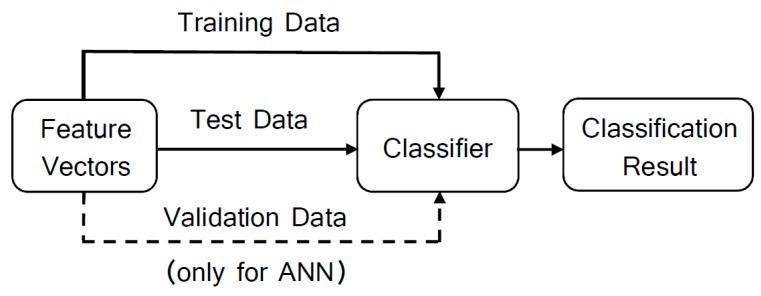
The signal classification.

Classifiers are used for pattern recognition, whose models are obtained through machine training using the feature vectors and their respective classes (or states). We executed a total of 40 weld beads. Of these, 196 features vectors were labeled in desired weld bead (class 1), shrinkage cavity (class 2) and burn through (class 3) as shown in Table 1. These features fed the classifier as shown in Figure 6. The discontinuities were classified according to the standard ISO 5817. We implemented the classifiers in a personal computer with CPU Corei 5 2.7 GHz, OS macOS Sierra, RAM 8GB, using MATLAB 2016a.

#### 2.3.1. Support Vector Machine

This work adopts the Support Vector Machine (SVM), which corresponds to one of the most successfully classifier employed for machine learning applications [24]. Basically, the classifier training consists in the determination of hyperplanes to separate data into classes. Given the total feature vectors, 135 feature vectors were used to train the SVM model and the remaining 61 vectors were employed for testing (Figure 6). For training we applied the linear type kernel, considering the constraint parameter C as unitary, together with the One-vs-All (OVA) binarization method.

The error in the training stage of the classifier can be minimized with the use of a high-quality training data set, and by increasing the parameter C. However, it is worth mentioning that increasing the constraint parameter reduces the generalization capacity, which is not desirable for the classifier. To evaluate the SVM performance, we computed the overall accuracy from the confusion matrix. This matrix associates the labels of the classes with the rows, and the columns with the measured data classification. The general accuracy computes the ratio of the sum of the diagonal elements, which correspond to the correct decisions of the classifier, with respect to the total elements of the matrix.

#### 2.3.2. Artificial Neural Networks

To compare the results obtained with of different classifiers, we propose a Back Propagation (BP) Artificial Neural Network (ANN) with one hidden layer to predict the weld bead class. This is a pattern recognition network with a feedforward network trained to classify inputs according to target classes. The input variables and ranges used for the model are the features vectors. Each input feature vector is propagated forward through the network until it reaches the output layer. The network has tree output neurons, which represent the three classes. In the output layer, only one of the three neurons should produce a 1, and the other neurons should output a 0.

In order to configure the ANN, we used 117 feature vectors for training the classifier model, 39 vectors were used to validate the network and to stop training before overfitting, and 40 vectors to test. The input and target vectors are randomly divided into these three sets. In the training step, the weight and bias values are updated according to the scaled conjugate gradient method. To evaluate the network performance, we computed the overall accuracy from the test confusion matrix.

## 3. Results and Discussion

This section describes the experimental results. A photo of a weld bead, as well the macrographs of its sections are introduced for discussion. Some signals from the experiment are presented. Results obtained with the SVM and ANN classifiers are presented.

### 3.1. Weld Bead Characteristics

**Figure 7:**
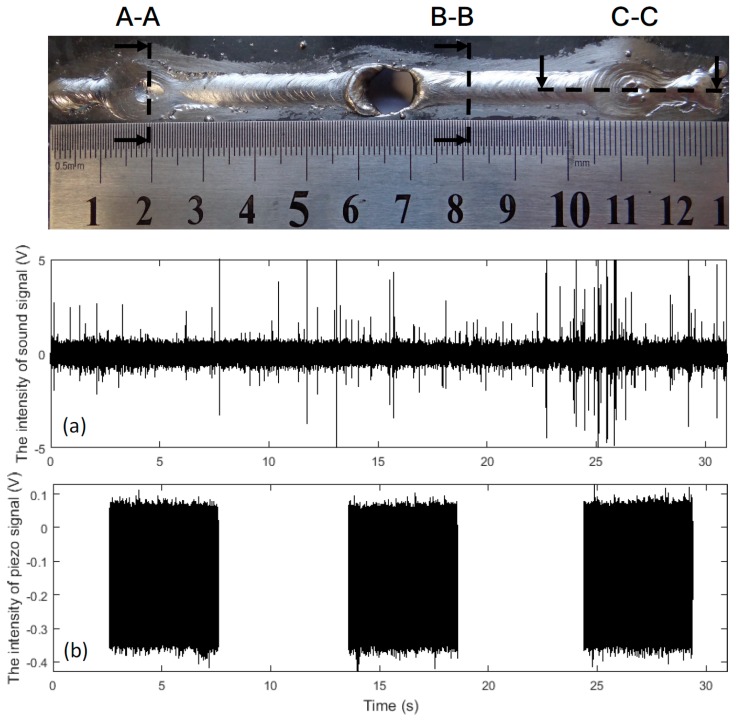
Typical weld bead obtained experimentally presenting the three classes: desired weld bead, shrinkage cavity and burn through. Cross-sectional A-A of a shrinkage cavity; cross-sectional B-B of the desired weld bead; longitudinal-sectional C-C of a shrinkage cavity. Signals collected by DAQs: (**a**) audible signal; and (**b**) acoustic emission in time.

In Figure 7 we have a typical weld bead obtained experimentally presenting the three classes studied. This bead was made with a current of 75 A, and an arc length of approximately 5 mm. This value was estimated after the electric arc recording analysis. Discontinuities occurred on the surface opposite the transversal slots (Figure 2). The intensity of the audible signal reduces slightly when the burn through discontinuity occurs. The sound signals related to shrinkage cavity discontinuities are discussed below.

**Figure 8:**
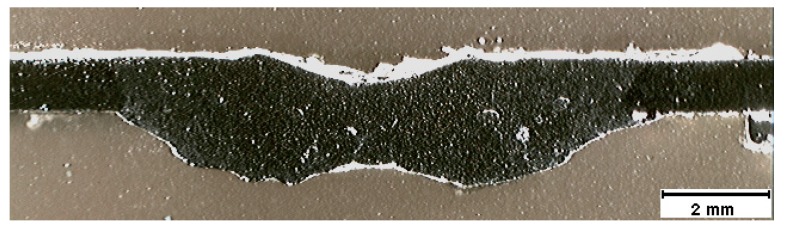
Macrograph (10×) of a shrinkage cavity. Cross-sectional A-A of the weld bead of Figure 7.

**Figure 9:**
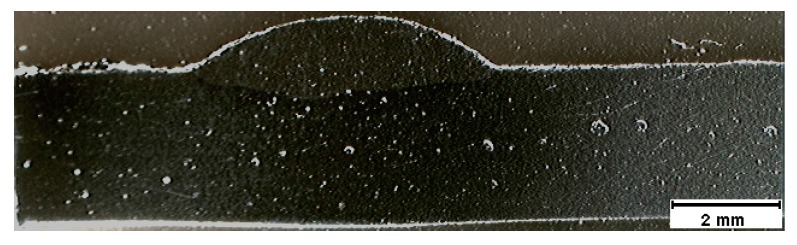
Macrograph (10×) of a desired weld bead. Cross-sectional B-B of the weld bead of Figure 7.

A macrograph of the cross-section A-A, representing the shrinkage cavity, is shown in Figure 8. A very high dilution is observed in this figure, which is not desired in weld beads, as it significantly changes the mechanical behavior of the material. The shrinkage cavity occurs due to excessive penetration and corresponds to the pre-burn through stage, which is observed at the second bead discontinuity. Figure 9 shows the cross-section B-B with the macrograph of the desired weld bead. The dimensions of this bead were width of 5.69 mm, height of 1.04 mm and penetration of 0.49 mm. The desired bead penetration was small, due to the short length of the arc [14]. The weld beads made with 70 A differ mainly because their beads have narrower widths.

**Figure 10:**
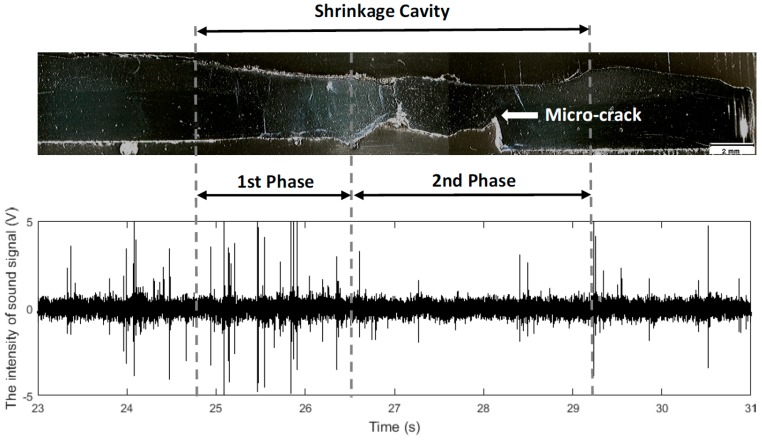
Macrograph (10×) of a shrinkage cavity (longitudinal-sectional C-C of the weld bead of Figure 7) and the correspondent audible signal collected by DAQ NI 9234.

A macrograph of the longitudinal section C-C is shown in Figure 10. Note that when the steel plate is reduced, the arc pushes the liquid metal beyond the lower surface of the base metal [28] and the arc length increases. As a result, the welding energy decreases, and the shrinkage cavities are formed during solidification. This phenomenon was observed in the recording of the weld pool. An important observation is that the audible signal changes with the shape of the weld pool during the shrinkage cavity formation. In the first phase, due to the increase in the arc length, the intensities of the audible signals vary significantly. After, as the melted materials decreases, the sound intensity becomes weaker.

**Figure 11:**
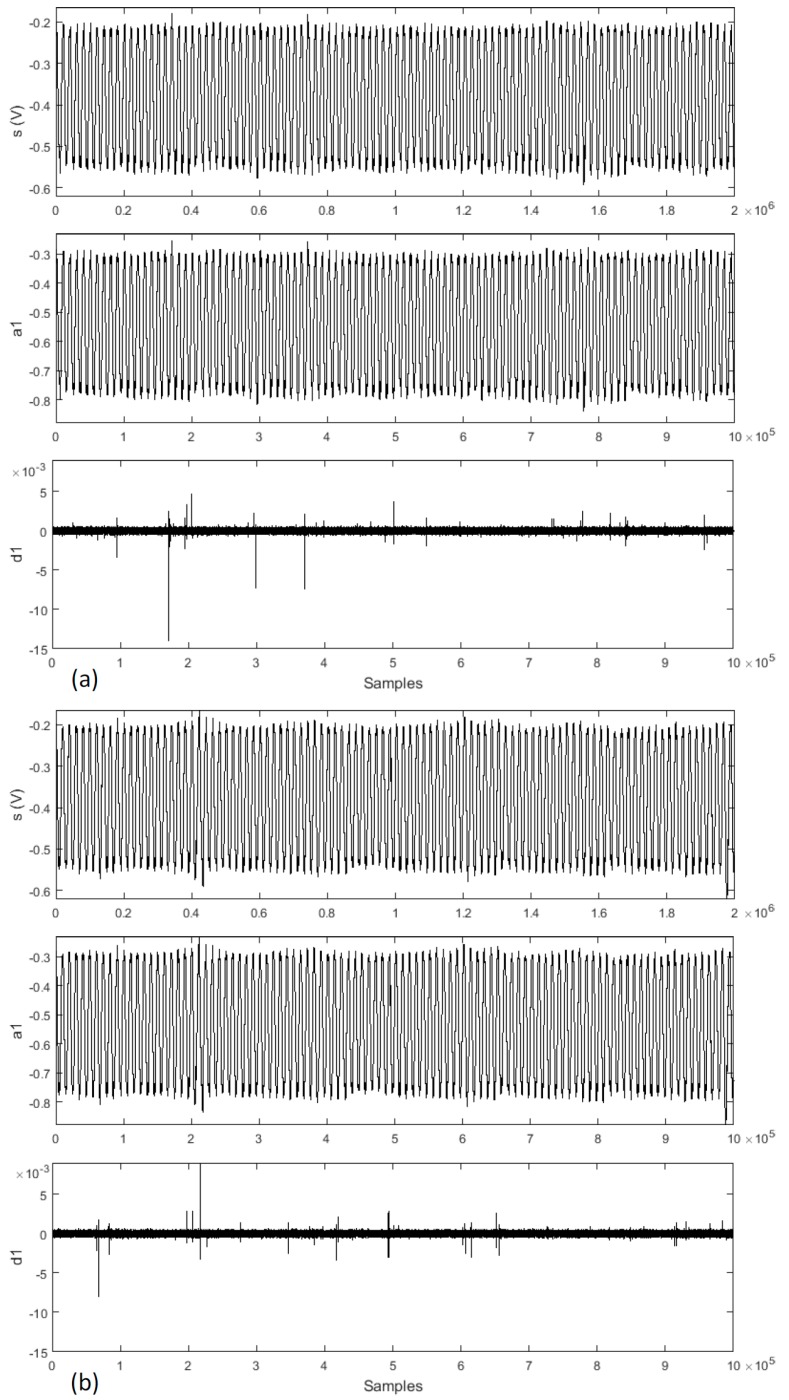
Wavelet descomposition of the piezoeletric signal, compared with shrinkage cavity (**a**) and burn through (**b**) discontinuities.

The wavelet decompositions of the signals from the welds with a shrinkage cavity and a burn through discontinuities are shown in Figure 11. Comparing the two sets of wavelet decompositions, it is possible to see that the intensity of shrinkage cavity approximation and detail are quasi-equal to the burn through approximation and detail. It is not possible to visually identify any difference. We applied the root mean square (RMS) function, and the obtained difference between the signals has order of 2%. In both discontinuities, the change of the weld bead shape occurs due to the lack of material.

Root micro-cracks are frequently found in the region where the thickness of the plate increased (Figure 10). Other discontinuities were observed in the experiments as well. In the shrinkage cavities, gas, surface and root pores are commonly found. This happens because the weld pool solidification rate is so fast that there is not enough time for the gases formed during the process to escape [29]. There are undercuts on the surface opposite to micro-cracks (2rd phase of Figure 10), which are caused by the lack of material. The presence of spatters is not significant in the experiments. The discontinuities observed in the shrinkage cavities as well the micro-cracks and spatters are not investigated in this research.

### 3.2. Classification

#### 3.2.1. Support Vector Machine

Among the 61 feature vectors used for the SVM tests, 34 corresponded to class 1, 17 to class 2, and 10 to class 3. These data were fed as input for the classification model, trained with feature vectors extracted from the microphone. For this classifier the accuracy is in the range of 80.3% to 95.1%, depending on the choice of seed in the training stage, with a median of 86.9% for the first 10 seeds. The best result of this classifier is presented in Table 2.

**Figure 12:**
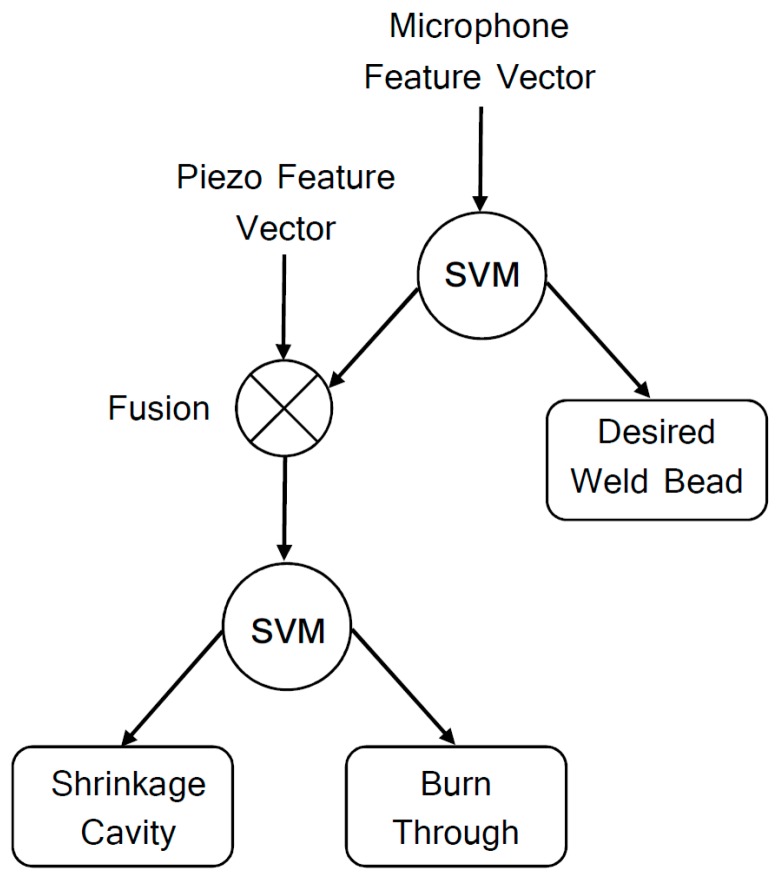
Hierarchical Support Vector Machine (HSVM) proposed for discontinuities classification in the weld bead for SMAW process.

The second classification model evaluation by SVM is performed in two stages. The structure of this classifier is hierarchical (HSVM), as shown in Figure 12. This is a novel HSVM structure, presenting two feature inputs in different stages. In the first stage, microphone data are evaluated whether or not they correspond to feature vectors of the desired weld bead. In negative case, a second stage of evaluation will be carried out to classify the discontinuity. In this case, the classifier model input will be fed with sensory fusion vectors, which consist of grouped vectors of the microphone and piezo sampled signals. The accuracy of this second proposed model is in the range of 84.9% to 96.6%, depending on the seed, with a median of 91.8%. The best result of this classifier is presented in Table 3 and Table 4.

All classes presented high classification accuracy, above 90%, for both proposed classifier structures. The classification results validate that the proposed models can be used to identify the three proposed classes. These models contain signals of time and frequency features that were collected by two different sensors. As the quantity of data raised with the use of sensory fusion, the overall accuracy increased from 95.1% to 96.6%. The robustness of the classifier also increased, as evidenced by the more expressive increase of the median overall accuracy.

#### 3.2.2. Artificial Neural Networks

Many attempts were carried out to choose the structure for the neural network, with 10 neurons in one hidden layer. Among the 40 feature vectors used for the ANN tests, 22 corresponded to class 1, 12 to class 2, and 6 to class 3. For the network trained with feature vectors extracted from the microphone, the accuracy is in the range of 75% to 97.5%, depending on the seed selection in the training stage, with a median of 83.8% for the first 10 seeds. The best result of this classifier is presented in Table 5.

In this model, 65 epochs minimized the error for the best result of this network. In this case, all classes presented accuracy above 90%. Because the overall accuracy median is worse than the one achieved with SVM (Table 6), the hierarchical ANN structure with sensor fusion was not implemented. However, it is important to mention that this network can be a workable model for prediction of the three proposed classes.

#### 3.2.3. Classification Discussion

Table 6 show the run-time results obtained in these three cases. Each segmented database corresponds to 1660 ms of recorded data. In Molino et al.’s [30] research, to detect lack of welding penetration and porosity defects in laser welding, it was necessary to process the data in less than 125 ms to ensure real-time computation.

As show in Table 6, segmented database could be preprocessed and classified in real-time computation for ANN and SVM classifiers. The training and classification times are sensitive to changes in neural network structure (numbers of hidden layers and neurons in each hidden layer). The SVM does not present this sensitivity. The HSVM structure proposed is feasible for real-time applications. It is a robust system to deal with disturbances and preprocesses signals with different dimensions in two different stages. In the first stage, the period for feature extraction is equal to that of the ANN and SVM classifiers. At this stage the weld bead is monitored looking for discontinuities. If the classifier diagnoses a failure, the second stage of HSVM is triggered. In this case, the extraction of sensory fusion characteristics will occur. This is the main advantage of the proposed strategy, increasing the computational cost only when it is necessary. A faster classifier could be implemented in other programming languages, such as C.

## 4. Conclusions

In this paper, pattern recognition techniques have been successfully applied for discontinuity detection in the SMAW process. SVM and ANN structures are configured to classify the weld beads, based on feature vectors from microphone data. After training, both structures can be used to identify the three proposed classes, which include desirable weld beads, shrinkage cavities and burn through discontinuities. The inspection accuracy for each class is greater than 90%. The acquired results suggest that both classifiers could be used for real-time applications. To implement the proposed techniques in industrial environments, a sensory fusion is performed by associating signals measured by microphone and piezoeletric sensors. For this purpose, we propose a novel HSVM structure with two feature inputs in stages. This approach presents 96.6% of overall accuracy.

It was shown in this work that the audible signals from burn through discontinuities in SMAW have significant high-order harmonics. This is the principal difference in frequency distributions compared to the other two classes. The similar classification results obtained in this work with different classifiers confirms that the feature extraction methodologies adopted are powerful tools for this application. The use of FFT with Average Downsampling to extract features from audible signal is particularly attractive due the relatively high speed available to execute the task.

It should be mentioned that, although the feasibility of the proposed HSVM has been verified, the system performance can be improved. Changing the base metal slot dimensions can be proposed to analyse if other discontinuities may appear. The heat input control can be tested to avoid discontinuities. Besides, other internal imperfections like cracks, porosity, incomplete fusion and lack of penetration should be considered in future works. Further studies concerning classification of gas pores, surface pores and root porosity discontinuities are in progress. Finally, the system is based on components widely available in the market, it can be extended to include other sensors, and it can be applied to pattern recognition in other arc welding processes.

## Figures and Tables

**Table 1:** Data of the Welds (* no data from piezo).

Class No.	Description	Number of Feature Vectors	
		Microphone	Piezo
1	Desirable weld	110	*
2	Shrinkage cavity	56	56
3	Burn through	30	30

**Table 2:** Classification Performance of SVM without Sensor Fusion.

Class No.	Description	Classified as # 1	Classified as # 2	Classified as # 3	Correct (%)	Incorrect (%)
1	Desirable weld	32	2	0	32 (94.2)	2 (5.8)
2	Shrinkage cavity	1	16	0	16 (94.2)	1 (5.8)
3	Burn through	0	0	10	10 (100)	0 (0)

**Table 3:** Classification Performance of SVM With Sensor Fusion–First Stage.

Class No.	Description	Classified as # 1	Classified as # 2&3	Correct (%)	Incorrect (%)
1	Desirable weld	33	1	33 (97.1)	1 (2.9)
2&3	Discontinuity	1	26	26 (96.3)	1 (3.7)

**Table 4:** Classification Performance of SVM with Sensor Fusion–Second Stage.

Class No.	Description	Classified as # 2	Classified as # 3	Correct (%)	Incorrect (%)
2	Shrinkage cavity	16	0	16 (100)	0 (0)
3	Burn through	1	9	9 (90)	1 (10)

**Table 5:** Classification Performance of ANN without Sensor Fusion.

Class No.	Description	Classified as # 1	Classified as # 2	Classified as # 3	Correct (%)	Incorrect (%)
1	Desirable weld	22	0	0	26 (100)	0 (0)
2	Shrinkage cavity	0	11	1	11 (91.7)	1 (8.3)
3	Burn through	0	0	6	6 (100)	0 (0)

**Table 6:** Run-time and Overall Accuracy of the Classifiers (* processing time for fusion feature vector of data from microphone and piezoelectric).

Classifier	Mean Run-Time for Each Segmented Database			Overall Accuracy (%)	Overall Accuracy Median (%)
	Feature Extraction	Training	Classification		
ANN	81	20.8	0.9	97.5	83.8
SVM	81	24.8	0.6	95.1	86.9
HSVM	325 *	156.9 *	2.4 *	96.6	91.8
